# Unexpected involvement of staple leads to redesign of selective bicyclic peptide inhibitor of Grb7

**DOI:** 10.1038/srep27060

**Published:** 2016-06-03

**Authors:** Menachem J. Gunzburg, Ketav Kulkarni, Gabrielle M. Watson, Nigus D. Ambaye, Mark P. Del Borgo, Rebecca Brandt, Stephanie C. Pero, Patrick Perlmutter, Matthew C. J. Wilce, Jacqueline A. Wilce

**Affiliations:** 1 Biomedicine Discovery Institute, Department of Biochemistry and Molecular Biology, Monash University, Wellington Road, Clayton VIC 3800, Australia; 2 School of Chemistry, Monash University, Wellington Road, Clayton VIC 3800, Australia; 3 Department of Surgery and Vermont Cancer Center, University of Vermont, Burlington 05401, USA

## Abstract

The design of potent and specific peptide inhibitors to therapeutic targets is of enormous utility for both proof-of-concept studies and for the development of potential new therapeutics. Grb7 is a key signaling molecule in the progression of HER2 positive and triple negative breast cancers. Here we report the crystal structure of a stapled bicyclic peptide inhibitor G7-B1 in complex with the Grb7-SH2 domain. This revealed an unexpected binding mode of the peptide, in which the staple forms an alternative contact with the surface of the target protein. Based on this structural information, we designed a new series of bicyclic G7 peptides that progressively constrain the starting peptide, to arrive at the G7-B4 peptide that binds with an approximately 2-fold enhanced affinity to the Grb7-SH2 domain (*K*_D_ = 0.83 μM) compared to G7-B1 and shows low affinity binding to Grb2-, Grb10- and Grb14-SH2 domains (*K*_D_ > 100 μM). Furthermore, we determined the structure of the G7-B4 bicyclic peptide in complex with the Grb7-SH2 domain, both before and after ring closing metathesis to show that the closed staple is essential to the target interaction. The G7-B4 peptide represents an advance in the development of Grb7 inhibitors and is a classical example of structure aided inhibitor development.

Protein-protein interactions are attractive targets for therapeutic intervention owing to their intrinsic role in many, if not most, cellular biological processes. Unlike small ligand binding sites, they offer large molecular surfaces that may be exploited for the development of highly specific inhibitors^1^. Accordingly, peptide or peptide-like molecules serve extremely well as inhibitors of protein interfaces as they can mimic the protein partner interface in terms of both interaction surface area and chemical functionalization^2^,^3^. Once a peptide inhibitor is developed with both potency and specificity for a target, it becomes an invaluable tool for investigating the biological significance of the interaction, and potentially for the development of new therapeutics targeting that interaction. There is hence a rising interest in the development of peptide-based inhibitors of protein-protein interactions^4^,^5^.

The process of inhibitor development, however, is rarely straightforward. After a lead molecule is discovered, by either a rational or a library-screening approach, the inhibitor must be optimised for potency and specificity. This is usually an iterative process that involves testing a series of systematically varied inhibitors, preferably guided by a structural understanding of the interaction. This, so called, “rational” approach is dependent upon our ability to predict molecular interactions and improvements to them. Herein, for all our computational power and improvements to free energy expressions, inhibitor design remains an uncertain science^6^. Each new inhibitor must be rigorously tested for its binding affinity and specificity for its target and, furthermore, its mode of binding cannot be assumed but must be verified by structure determination. Only in this way can a rational approach to inhibitor development progress.

The importance of structural characterisation is illustrated in the current study that describes progress in the development of peptide inhibitors targeted to Grb7. Growth factor receptor-bound protein 7 (Grb7) is an adaptor protein involved in cell signaling where it interacts with a number of tyrosine kinases via its src-homology2 (SH2) domain^7^,^8^. In this way it plays a role in integrin dependant cell migration via its interaction with focal adhesion kinase and subsequent phosphorylation in focal contacts, consistent with its homology to the *C. Elegans* protein Mig-10 that is involved in cellular migration^9^,^10^. It has been shown that overexpression of Grb7 enhances cell migration, while inhibition of Grb7 lowers the migratory potential of cells and is therefore linked with metastatic spread of cancer cells^11^. Grb7 also interacts with the ErbB2/3 receptor and is co-overexpressed with ErbB2 in a number of breast cancer cell lines, primary breast tumors^12^ and in esophageal and gastric carcinoma^13^,^14^. It is thus also implicated in cell proliferation and cells survival in cancer^15^,^16^. While co-overexpression of Grb7 and ErbB2 occurs due to the proximity of their two genes on the 17q12-21 amplicon, Grb7 plays a role independent of ErbB2 in cancer progression^17^,^18^,^19^. These important roles of Grb7 in numerous cancers have established Grb7 as a therapeutic target^20^,^21^,^22^.

The non-phosphorylated peptide G7-18NATE (cyclo-(CH_2_CO-WFEGYDNTFPC)-amide), identified via a phage display, inhibits Grb7 interactions with ErbB3 and FAK in cell lysates and represents an important lead compound that targets events upstream of Grb7 signalling^23^. G7-18NATE with an additional cell permeability sequence inhibits the growth of a number of breast cancer cell lines, yet has no effect on non-malignant cells and is synergistic with chemotherapeutics Doxorubicin and Trastuzumab, reducing their EC_50_ values^24^,^25^. In another study G7-18NATE was shown to significantly attenuate cell migration and reduce metastasis in a human pancreatic cancer mouse model^24^,^25^. In the presence of phosphate G7-18NATE possesses a high degree of specificity for Grb7-SH2 domain over related Grb2-, Grb10- and Grb14-SH2 domains^26^. Binding to Grb7-SH2 occurs with only a *K*_D_ = 4.1 μM, however, so higher affinity ligands are sought to improve the potential uses of this peptide. One strategy has been to replace the tyrosine with carboxymethylphenylalanine or carboxyphenylalanine as a phosphotyrosine mimetic, enhancing the binding affinity 9-fold under physiologically relevant conditions^27^. A second strategy involves rigidification of the G7-18NATE cyclic framework. The crystal structure of the Grb7-SH2 domain in complex with G7-18NATE revealed that adjacent W1 and T8 sidechains in G7-18NATE do not play a direct role in the interaction with Grb7-SH2 domain^28^. Subsequently a bicyclic derivative to G7-18NATE, with an O-allylserine-based olefin linkage (from herein referred to as a staple) between positions 1 and 8 (named G7-B1), was shown to have a 2–3 fold higher affinity for Grb7^29^.

Here we report the crystal structure of G7-B1 in complex with Grb7-SH2 domain, revealing an unanticipated mode of binding involving interactions between the staple and Grb7-SH2 domain. This finding was used to subsequently design increasingly constrained derivatives of G7-B1. We show that the G7-B4 peptide binds with an improved binding affinity of *K*_D_ = 0.83 μM and retains specificity for Grb7-SH2 domain over Grb2, Grb10 and Grb14-SH2 domains. Furthermore we have solved the structure of the G7-B4 peptide in complex with Grb7-SH2 domain, both before and after ring closure metathesis, to show that the staple is essential to the target interaction. These studies establish the structural basis of binding by a new peptide lead and demonstrate the importance of structural insight to the process of inhibitor development.

## Results

### Crystal structure of the Grb7-SH2/G7-B1 complex reveals the involvement of the staple

**Figure 1:**
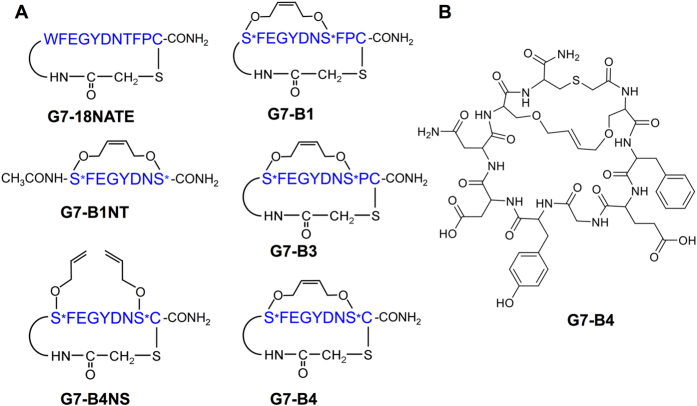
Schematic showing the structures of the 6 peptides referred to in this paper. (**A**) Schematic depicts amino acid single letter code in blue, with S* referring to O-allylserine. Other chemical functionalities are depicted in black. (**B**) chemical structure diagram of the G7-B4 peptide.

We have previously shown that the cyclic peptide G7-18NATE has a 2–3 fold improved affinity for the Grb7-SH2 domain when modified to incorporate an O-allylserine derived staple between residues 1 and 8 (termed G7-B1; Fig. 1)^29^. The G7-B1 peptide was designed to rigidify the Grb7-SH2 binding conformation observed for G7-18NATE and thereby enhance affinity by reducing entropic loss upon binding. This appeared to be achieved when tested using isothermal titration calorimetry^29^. However, to comprehensively determine the structural basis for the improved affinity of G7-B1 over G7-18NATE we solved the structure of the Grb7-SH2/G7-B1 complex using X-ray crystallography. The preparation of complex crystals, data collection and data collection statistics have been reported previously^30^.

The Grb7-SH2/G7-B1 crystal structure was solved to 1.6 Å resolution using a monomer of apo-Grb7-SH2 as the molecular replacement search model (PDB ID: 2QMS). The refinement statistics are provided in Table 1 and the coordinates are deposited in the RCSB database (PDB: 5EEQ). The asymmetric unit consists of two Grb7-SH2 domains (with residues 426–528 in chain A and 424–527 in chain B visible in the electron density), two G7-B1 peptides, 171 water molecules, and two phosphate anions that are positioned in the phosphotyrosine binding site of each Grb7-SH2 monomer.

**Figure 2:**
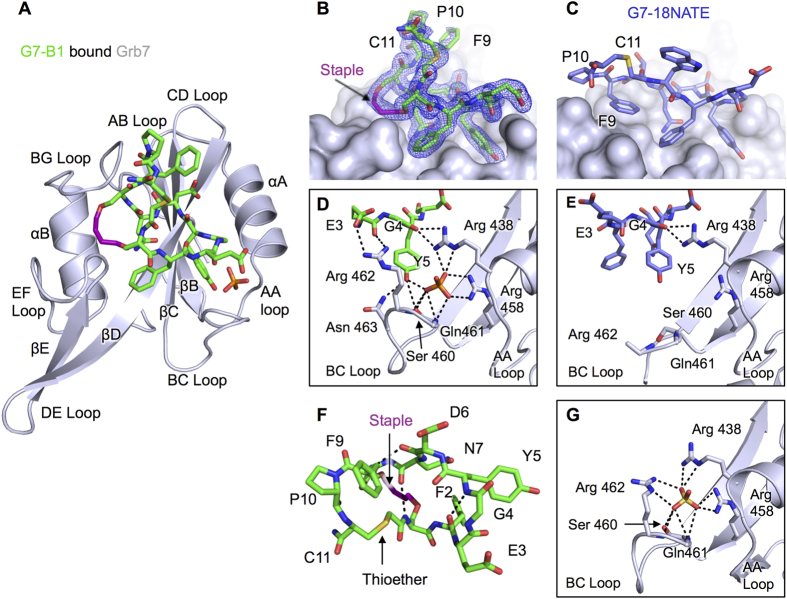
Structure of the G7-B1 and Grb7-SH2 domain complex in comparison to other Grb7-SH2 structures. (**A**) Cartoon representation of the Grb7-SH2 domain (grey), with G7-B1 (green) and phosphate ion in stick representation. (**B**) G7-B1 peptide shown in green stick representation at the surface of Grb7-SH2 domain (grey), with 2Fo-Fc map surrounding G7-B1 shown contoured at 1.2σ. (**C**) G7-18NATE (purple sticks) bound to Grb7-SH2 domain (grey surface) (PDB ID: 3PQZ) in the same orientation as in (**B**). (**D**) G7-B1 (green sticks) oriented to show intramolecular hydrogen bond and electrostatic interactions at the pY binding pocket of Grb7-SH2 (grey cartoon). (**E**) Grb7-SH2 domain (grey cartoon) pY binding pocket interactions with G7-18NATE (purple sticks) (PDB ID: 3PQZ) in the same orientation as in (**D**). (**E**) G7-B1 structure (green sticks) shown in absence of Grb7-SH2 binding partner to highlight intramolecular hydrogen bonds. (**G**) apo-Grb7-SH2 domain pY binding pocket (grey cartoon) showing bound sulfate (PDB ID: 2QMS).

The Grb7-SH2 domain adopts the typical SH2 fold (Fig. 2A) as previously observed for the apo-structures of the Grb7-SH2 domain and the structure of Grb7-SH2 bound to the G7-18NATE peptide^28^,^31^. Following the conventional nomenclature for SH2 domains^32^, the Grb7-SH2 domain possesses a 3 stranded anti-parallel β-sheet consisting of the βB, βC and βD strands, flanked by 2 α-helices, αA and αB (Fig. 2A). In addition, the Grb7-SH2 domain possesses a βE strand, which extends the central β-sheet. The Grb7-SH2 chains within the asymmetric unit form a dimer in which the interface is formed by the packing of the two αB helices, including stacking of opposing Phe511 aromatic rings, and hydrogen bonds between the Arg501 backbone NH and CO and Asn 515 sidechain OD1 and ND2 respectively (note that three letter code will be used in referring to protein amino acid residues, while single letter code will be used to refer to peptide ligand amino acids, in order to clearly distinguish them from each other). This dimer interface is observed in all previously solved structures of the Grb7-SH2 domain^27^,^28^,^31^. Overall, the C^α^ RMSD between residues 426–526 is 0.58 Å compared with the Grb7-SH2 domain from the Grb7-SH2/G7-18NATE complex.

The G7-B1 peptide, however, did not adopt the expected structure when bound to the Grb7-SH2 domain. Figure 2B shows the structure of G7-B1 within the binding pocket of the Grb7-SH2 domain. The position of the cyclic peptide backbone and the extra cycle formed by the staple is clearly defined by the electron density. The immediate striking feature of the G7-B1 interaction with the Grb7-SH2 domain is that, instead of the staple acting as a stabilising tether, it acts to form a new Grb7-SH2 domain interaction surface. While the amino acid residues 2–7 are positioned as expected from our knowledge of the G7-18NATE interaction (Fig. 2C), residues 9, 10 and 11 no longer interact at the surface of the protein but form a loop that sits above the staple away from the protein surface.

Residues 2–7 of the G7-B1 peptide form the main binding interactions within and near the phosphotyrosine (pY) binding pocket of the Grb7-SH2 domain (Fig. 2D). Y5 is positioned in the pY binding site precisely as seen in the G7-18NATE structure (Fig. 2E) forming hydrogen bonds with Asn463(ND2) and Ser460(OG) in the BC loop of Grb7-SH2 (Fig. 2D). Additionally the Y5 of G7-B1 forms a hydrogen bond with the phosphate anion bound to the Grb7-SH2 domain, together effectively mimicking the presence of a pY. The phosphate anion, in turn, forms hydrogen bonds with core binding pocket residues including Arg438(NH2 and NE), Arg458(NH1 and NH2), Ser 460(OG) and Gln 461(NH) similar to interactions seen in the Grb7-SH2 apo-structure PDB:2QMS (Fig. 2G). Similar to the previously solved structure for G7-18NATE bound to Grb7-SH2, the G4 carbonyl oxygen hydrogen bonds with the Arg438 sidechain (NH1 and NH2) (Fig. 2E,F) while D6(NH and OD2) form hydrogen bonds with the main chain of His 479(CO and NH). N7(ND2 and OD1) form hydrogen bonds with the main chain of Met 495(CO) and Leu 481(CO and NH). G7-B1 is further stabilised by hydrogen bonds and a salt bridge between E3(OE1 and OE2) and Arg 462(NH1 and NH2) with these interactions not previously seen in Grb7-SH2/peptide structures (Fig. 2D,E). The intramolecular hydrogen bonding for G7-B1 is similar to G7-18NATE, with a β-turn formed between F2(CO) and Y5(NH) (Fig. 2F)^27^,^28^. Additionally a β-turn is observed between D6(CO) and F9(NH) and a hydrogen bond is formed between N7(CO) and allylS1(NH) across the centre of the ring.

In contrast to the G7-18NATE bound structure, residues F9, P10 and C11 are no longer positioned against the EF and BG loop surfaces of the Grb7-SH2 domain^28^. Instead the staple forms close contacts with Met 495, Asp 496, Asp 497 mainchain and sidechain atoms in the EF loop of Grb7-SH2 and Ile 518 sidechain in the BG loop (Fig. 2A). The staple contributes 83 Å^2^ of the total interaction surface area of 548 Å^2^. F9, P10, C11 and the thioether linkage are solvent exposed with no buried surface area. Thus the arrangement of these two loops in the bicyclic peptides are inverted with respect to their expected arrangement, with the F9, P10, C11 tripeptide acting as a tether across the stapled peptide rather than vice versa. This suggests that the higher affinity reported for the G7-B1 peptide (*K*_D_ = 1.9 μM)^29^ compared to that of G7-18NATE (*K*_D_ = 4.1 μM) arises from this alternate peptide configuration that places the staple at the interaction interface rather than the anticipated conformation with restriction imposed by the staple.

### The thioether linkage is required for peptide binding to Grb7-SH2

The Grb7-SH2/G7-B1 crystal structure shows that, while the staple interacts with the Grb7-SH2 domain, the F9, P10, C11 and the thioether linkage no longer play a direct role in peptide binding. We therefore synthesized a peptide without residues 9–11 (and hence without the thioether linkage), designated G7-B1NT (for G7-Bicyclic 1 No Thioether), in order to determine whether these residues and the thioether could be removed from the peptide without affecting its binding to the Grb7-SH2 domain. Binding experiments were conducted for G7-B1 and G7-B1NT using SPR under the same conditions as previously used for the determination of G7-18NATE and G7-B1 binding (using ITC) to the Grb7-SH2 domain^26^,^29^.

**Figure 3:**
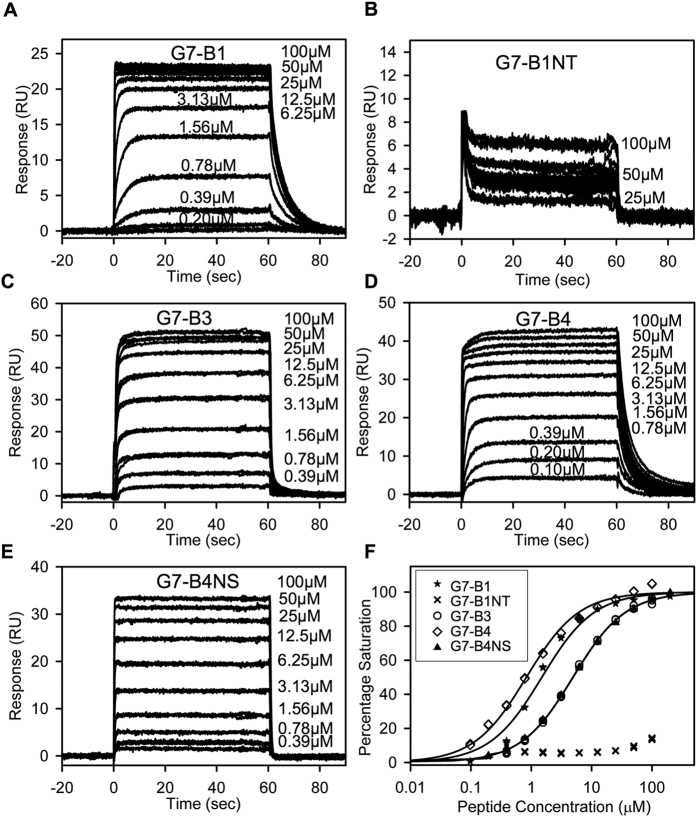
Binding affinity determination of G7 peptides for Grb7-SH2. (**A–F**) SPR Sensorgrams for G7 peptides (0–100 μM) binding to the Grb7-SH2 domain. The peptide samples were injected from 0 to 60 s; otherwise, buffer was flowing. (**A**) G7-B1. (**B**) G7-B1NT. Labels for low peptide concentrations are omitted for clarity. (**C**) G7-B3. (**D**) G7-B4. (**E**) G7-B4NS. (**F**) Equilibrium binding curves for peptides binding to the Grb7-SH2 domain (shown using a logarithmic scale for clarity). Percentage saturation for G7-B1NT was calculated as B_max_ of 0.64 × Theroretical B_max_ (based on the average B_max_/Theroretical B_max_ ratio for all other peptides). Fits to a single-site binding model are shown as solid lines. Data for G7-B4 served as a control in Fig. 4D.

Binding of the G7-B1 peptide to the Grb7-SH2 domain gave rise to sensorgrams showing that binding equilibrium was reached, allowing a steady-state equilibrium binding curve to be constructed for the determination of *K*_D_ (Fig. 3A). The equilibrium binding curve showed an excellent fit by a one-site binding model (R^2^ = 0.9850) with a *K*_D_ determined at 1.5 μM (Fig. 3F and Table 2), slightly lower than the value previously determined by ITC^29^. In addition, although the sensorgrams showed association-rate kinetics too fast to be fit by kinetic models, they did allow the off-rate of the interaction to be determined at ~*k*_d_ = 0.21 s^−1^.

In contrast, very weak binding was observed for the G7-B1NT peptide with a low response shown even upon application of G7-B1NT at 100 μM (Fig. 3B). The sensorgrams reveal very fast association and dissociation rates and thus too rapid to reliably fit with kinetic models. However, the interaction reached equilibrium within the timeframe, allowing the construction of a steady-state equilibrium binding curve (Fig. 3F). The equilibrium binding curve for G7-B1NT shows no curvature up to a concentration of 100 μM and the responses observed in the binding sensorgrams are much lower that the theoretical maximum binding response (Fig. 3B and Table 2) indicating that G7-B1NT binds too weakly to the Grb7-SH2 domain to determine a dissociation constant. This demonstrates that the thioether linkage, present in G7-B1, is essential for the observed strong binding affinity for the Grb7-SH2 domain.

### Shortening of the thioether-linked loop further enhances peptide binding affinity

Having established the importance of the thioether-linked tether, we next considered whether reducing it in length would have the effect of increasing the binding affinity of the G7-B1 peptide. This was anticipated to have the effect of constraining the peptide in the bound conformation and thereby enhancing its affinity – as was the original rationale for incorporating the staple^29^. We thus designed and synthesized two bicyclic peptides based on G7-B1, with F9 deleted (designated G7-B3) and with F9 and P10 deleted (designated G7-B4; Fig. 1A,B). The C-terminal cysteine was retained in these peptides in order to be able to form the thioether linkage.

The binding of G7-B3 and G7-B4 to Grb7-SH2 was measured using SPR under the same conditions used previously. The SPR sensorgrams again show association and, in the case of G7-B3, dissociation kinetics that are too fast to be fit by kinetic models (Fig. 3C). In both cases equilibrium was reached, however, allowing for the construction of steady-state equilibrium binding curves for the determination of *K*_D_. The equilibrium binding curve for G7-B3 binding to Grb7-SH2 showed an excellent fit by a one-site binding model (R^2^ = 0.9990) with a *K*_D_ determined at 4.9 μM (Fig. 3F and Table 2). This *K*_D_ represents an approximately 3 times weaker affinity compared to G7-B1. The SPR sensorgrams for G7-B4 binding to Grb7-SH2 (Fig. 3D), in contrast to G7-B3, showed distinctly slower dissociation rates, indicative of an increased residence time of the peptide binding to Grb7-SH2. The off-rate could be measured and was found to be ~*k*_d_ =  0.25 s^−1^. The dissociation constant of G7-B4 binding to Grb7-SH2 determined from the equilibrium binding curve was determined at *K*_D_ = 0.83 μM, with an excellent fit to a one-site binding model (R^2^ = 0.9926) (Fig. 3F and Table 2). Together these experiments show that while the removal of the F9 residue from G7-B1 did not improve the binding affinity (and unexpectedly decreases it), the removal of both F9 and P10 resulted in a peptide with approximately twice the affinity for Grb7-SH2 compared to G7-B1 and with 5 times higher affinity than G7-18NATE^26^,^29^,^33^.

### The G7-B4 staple is required for high affinity binding to Grb7-SH2

To determine whether the fully formed staple (rather than just the O-allylserine functionalities) is required for the high affinity binding to the Grb7-SH2 domain we synthesized the G7-B4NS peptide (for G7-Bicyclic 4 No Staple; Fig. 1A) which included the thioether linkage to achieve cyclisation but was not subjected to ring-closing metathesis to form a staple via O-allyl substituted serine residues. G7-B4NS binding to the Grb7-SH2 domain showed a similar SPR sensorgram to G7-B3 with extremely fast association and dissociation rates that, again, could not be measured. (Fig. 3E). Equilibrium was reached rapidly and the calculated equilibrium binding curve gave excellent fits by a one-site binding model (R^2^ = 0.9993) (Fig. 3F and Table 2). The dissociation constant determined for G7-B4NS binding to Grb7-SH2 was *K*_D_ = 4.9 μM. This represents a 6-fold loss in binding affinity compared to G7-B4 showing that the staple is required for high affinity binding to the Grb7-SH2 domain.

### G7-B4 is specific for Grb7-SH2 compared to closely related SH2 domains

**Figure 4:**
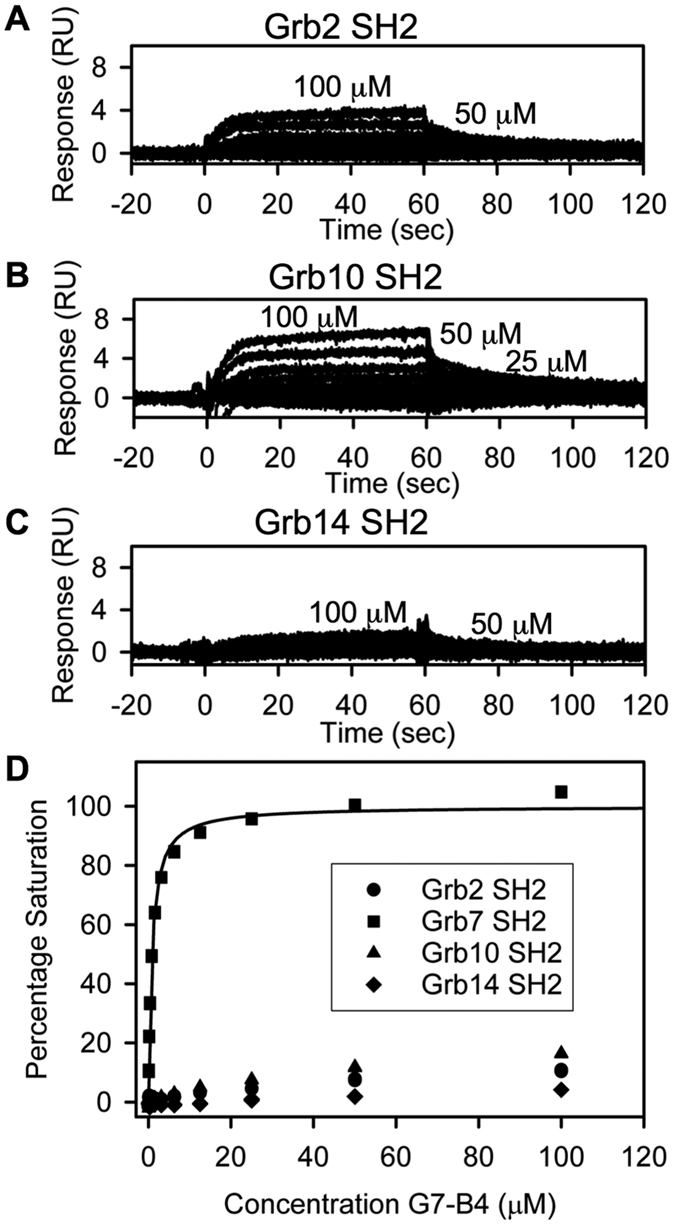
Determination of binding specificity of G7-B4 for Grb7-SH2. (**A–C**) Sensorgrams for G7-B4 (0–100 μM) binding to different Grb protein SH2 domains. The G7-B4 samples were injected from 0 to 60 s; otherwise, buffer was flowing. Labels for low G7-B4 concentrations are omitted for clarity. (**A**) Binding to Grb2-SH2 domain. (**B**) Binding to Grb10-SH2 domain. (**D**) Binding to Grb14-SH2 domain. (**D**) Equilibrium binding curves for G7-B4 binding to different Grb SH2 domains. Percentage saturation for Grb2-SH2, Grb10 SH2 and Grb14 SH2 were calculated as B_max_ of 0.64 × Theroretical B_max_ (based on the average B_max_/Theroretical B_max_ ratio for peptides binding Grb7). Fit to a single-site binding model is shown as solid line. Data for G7-B4 binding to Grb7-SH2 was also used in Fig. 3F.

Previous studies have established the preferential binding of the G7-18NATE peptide to Grb7-SH2 over closely related SH2 domains (Grb10- and Grb14-SH2 domains that share 67% and 65% identity respectively, to the Grb7-SH2 and Grb2-SH2 (~26% identity) domain that shares the pYXN peptide recognition motif)^26^. In order to determine whether the bicyclic peptide G7-B4 retains its specificity for the Grb7-SH2 domain we measured the binding of G7-B4 to Grb2-, Grb10- and Grb14-SH2 domains using SPR. The SPR sensorgrams for G7-B4 binding to Grb2-SH2, Grb10-SH2 and Grb14-SH2 (Fig. 4A–C) show very weak binding but could be used to derive equilibrium binding curves (Fig. 4D). The equilibrium binding curves for G7-B4 binding to Grb2-SH2, Grb10-SH2 and Grb14-SH2 show no curvature up to a concentration of 100 μM and the responses observed in the binding sensorgrams are much lower that the theoretical maximum binding response indicating that the peptide binds too weakly to the Grb2-, Grb10- and Grb14-SH2 domains for the determination of a dissociation constant (Fig. 4 and Table 2). The apparent percentage saturation for G7-B4 binding to Grb2-, Grb10- and Grb14-SH2 domains is well below 50% suggesting that the dissociation constants for these interactions must be >100 μM. Therefore the binding of G7-B4 to Grb2-, Grb10- and Grb14-SH2 is over 130 times weaker than binding to Grb7-SH2 domain, showing that G7-B4 is highly specific for the Grb7-SH2 domain.

### The structure of the Grb7-SH2/G7-B4 complex is similar to the Grb7/G7-B1 structure

In order to characterise the structural basis for the improved affinity of G7-B4 as compared to G7-B1 for Grb7-SH2 domain, we solved the structure of the Grb7-SH2/G7-B4 complex using X-ray crystallography to 2.47 Å resolution. The structure was solved in the P 4_1_2_1_2 space group using the previously determined apo-Grb7-SH2 (PDB ID: 4WWQ) as a molecular replacement model. The refinement statistics are provided in Table 1 and the coordinates are deposited in the RCSB database (PDB ID: 5EEL). The asymmetric unit consists of six Grb7-SH2 domains, six G7-B4 peptides, 75 water molecules, six malonic acid molecules and six formate molecules (present in the crystallisation conditions). Residues 428–529 of Grb7-SH2 domains are clearly visible in the electron density maps for all chains A-F. The Grb7-SH2 domain within the G7-B4 complex adopts a typical SH2 fold, without any major changes in structure compared to the Grb7-SH2 domain in complex with the G7-B1 peptide. This includes the occurrence of dimer formation via the packing of two αB helices. The C^α^ RMSD between G7-B1 and G7-B4 bound forms of Grb7-SH2 is 0.53 Å across residues 426–528. The most significant region of structural change is observed in the BC loop with slight differences in hydrogen bonding to the pY binding pocket ligand leading to an altered BC loop arrangement.

**Figure 5:**
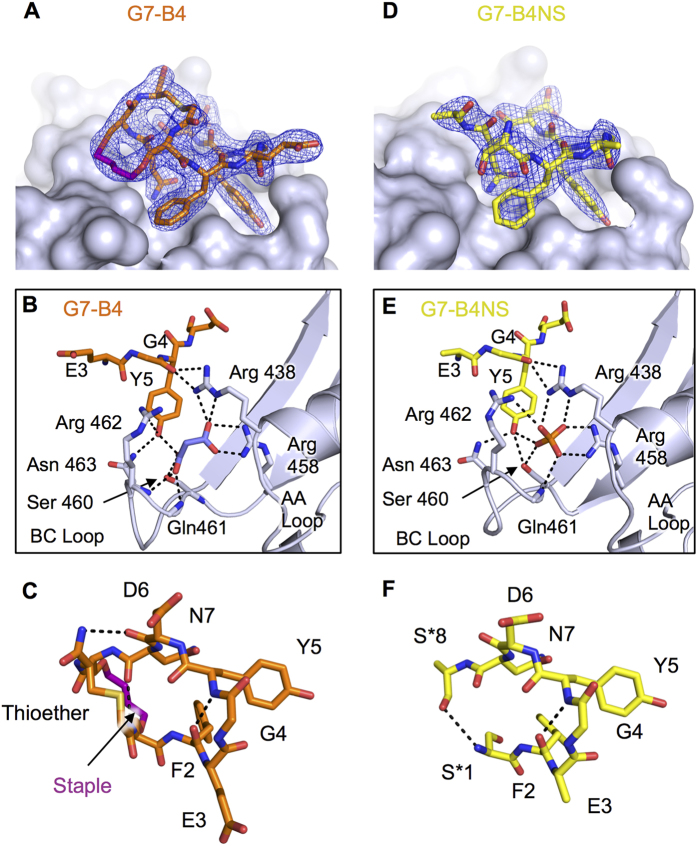
Structure of the G7-B4 and G7-B4NS peptide in complex with Grb7-SH2. **(A**) G7-B4 peptide shown in orange stick representation at the surface of Grb7-SH2 domain (grey), with 2Fo-Fc map surrounding G7-B4 shown contoured at 1.2σ. (**B**) Details of the G7-B4 bound pY binding pocket. G7-B4 and malonic acid (purple sticks) bound to Grb7-SH2. Key amino acids are shown as sticks and hydrogen bonds as dashed lines. (**C**) G7-B4 oriented to show intramolecular hydrogen bonds. (**D**) G7-B4NS peptide shown in yellow stick representation at the surface of Grb7-SH2 domain (grey), with 2Fo-Fc map surrounding G7-B4NS shown contoured at 1.2σ. (**E**) Details of the G7-B4NS pY binding pocket. G7-B4NS and phosphate bound to Grb7-SH2. Key amino acids are shown as sticks and hydrogen bonds as dashed lines. (**F**) G7-B4NS oriented to show intramolecular hydrogen bonds.

The bound G7-B4 peptide is clearly defined in the electron density, and shows that the staple is again engaged in interactions with the Grb7-SH2 domain (Fig. 5A). The staple forms close contacts with Met495, Asp496, Asp497 backbone and sidechains in the EF loop of Grb7-SH2 and Ile 518 in the BG loop in the same way as seen for G7-B1. The staple contributes 55 Å^2^ of the total interaction surface area of 424 Å^2^. Figure 5B shows the Grb7-SH2 pY binding pocket residues involved in the interaction with G7-B4. As was observed for G7-B1 (Fig. 2) Y5 of G7-B4 is located within the pY binding pocket and forms hydrogen bonds with Asn463(D2) and Ser460(OG) in the BC loop of Grb7-SH2. A hydrogen bond is again observed between G4(CO) and Arg438(NH1 and NH2). The G7-B4 structure, however, shows a loss of some close contacts that were present in G7-B1, including the loss of the salt bridges between E3 and Arg 462 in the BC loop, and the loss of a hydrogen bond between Y5(OH) and Ser460(OG). The Y5(OH) of G7-B4 also forms a hydrogen bond with a malonic acid that is bound in the Grb7-SH2 domain pY binding pocket, with the interaction occurring in a similar fashion to the phosphate interaction in the G7-B1 co-crystal structure (Figs 2E and 5B). The malonic acid, in turn, forms hydrogen bonds with Arg458(NH1 and NH2), Ser 460(OH) and Gln 461(NH) at the end of the βB and in the BC loop of Grb7-SH2 domain, in addition to Arg438(NE) in αA. Similar to the structure for G7-B1 bound to Grb7-SH2 a β-turn is formed with a hydrogen bond formed between F2(CO) and Y5(NH), another hydrogen bond is formed between N7 (CO) and O-allyl-S1(NH) and the hydrogen bond between D6(CO) and F9(NH) in G7-B1 is replaced by one between D6(CO) and C9(NH) in G7-B4 (Fig. 5C).

The thioether linkage is well defined in the electron density, positioned across the top of the stapled cyclic peptide, linking residues 1 and 8, but not altering their positions compared to those in the G7-B1 structure. The thioether linkage is solvent exposed with no buried surface area (except for making crystal contacts in the case of one out of six of the molecules in the asymmetric unit). The increase in affinity of G7-B4 for Grb7-SH2 domain over that of G7-B1 occurs despite the fact that there are no new intermolecular interactions formed between the peptide and protein (besides those that can be seen in the crystal structure due to the ion present in the crystallisation conditions) nor additional intramolecular hydrogen bonds. The only clear difference between the structures is that the thioether linker is shortened in G7-B4 compared to that in G7-B1.

### The structure of the G7-B4NS/Grb7-SH2 complex confirms that direct interaction of the staple contributes to the affinity of the peptide

To investigate the observation that the ring-closed G7-B4 peptide bound with 6 fold higher affinity than the G7-B4NS peptide that had not been subjected to ring-closing metathesis, we determined the structure of the Grb7-SH2/G7-B4NS complex using X-ray crystallography, to 2.6 Å resolution. The structure was solved in the P 2_1_2_1_2_1_ space group using apo-Grb7-SH2 (PDB: 2QMS) as the search model. The refinement statistics are provided in Table 1 and the coordinates are deposited in the RCSB database (PDB: 5D0J). The asymmetric unit consists of four Grb7-SH2 domains (consisting of residues 429–527), two G7-B4NS peptides, 3 water molecules, and two phosphate anions.

In this structure the Grb7-SH2 again domain adopts a typical SH2 fold, without any major changes in structure compared to Grb7-SH2 in complex with the G7-B4 peptide, with an C^α^ RMSD between G7-B4 bound form and G7-B4NS bound form of Grb7-SH2, of 0.89 Å across 101 residues. The most significant region of structural change is observed in the BC loop, and the DE loop, while all α helices and β-stands show minimal structural change (Fig. 5E).

Figure 5D shows the structure of the G7-B4NS within the binding pocket of the Grb7-SH2 domain. Consistent with the previous structures Y5 of G7-B4NS is located within the phosphotyrosine binding pocket and forms hydrogen bonds with Asn463(ND2) and Ser460(OG) in the BC loop of Grb7-SH2 (Fig. 5E). Additionally the Y5 of G7-B4NS forms analogous interactions to the Y5 of G7-B1 with the phosphate anion positioned in the pY binding pocket (Fig. 2D). The phosphate forms all the same interactions as previously observed with amino acid residues at the end of the βB, in the BC loop and in in αA of Grb7-SH2 domain in the G7-B1 co crystal structure. Likewise, the G7-B4NS peptide residues 2–7 all form the same interactions with Grb7-SH2 as observed in the G7-B4 co-crystal structure. Accordingly, the internal structure of bound G7-B4NS between residues 2–7 (Fig. 5F) is analogous to that of G7-B4 (Fig. 5C).

Electron density for the peptide, however, was not visible for the O-allylserine sidechains (and, in one molecule within the asymmetric unit, was not visible for the N-terminal two residues and, in the other molecule, not seen for the C-terminal cysteine). This absence of electron density suggests these groups are flexible and do not form an interaction with the surface of the Grb7-SH2 domain. There was also an absence of electron density for the thioether linker indicating that this linker is more flexible in this crystal form or in complex with Grb7-SH2 than the equivalent thioether linker observed in G7-B4. This suggests that the fully formed staple is required for forming interactions with the Grb7-SH2 domain.

## Discussion

Stapled peptides have become a major focus for their potential as potent inhibitors of protein-protein interactions^34^,^35^. By constraining the peptide structure to its bound conformation, staples are understood to increase peptide binding affinity through reducing the entropic penalty of forming a bound structure. In addition, stapled peptides are conferred with increased half-life *in vivo* and improved bioavailabilty^36^. In particular, staple formation via ring closing metathesis to form olefin-based staples has been utilised owing to its ease of incorporation into solid-phase peptide synthesis protocols^37^. While the most intensive efforts have exploited olefin-based staples for stabilisation of α-helical bioactive peptides^36^,^38^, this chemistry has also been applied to other peptide scaffolds, including cyclic peptides and in the replacement of disulphide bonds^39^,^40^.

The current study has utilised ring closing metathesis of O-allylserine residues to staple the cyclic peptide G7-18NATE targeted to the SH2 domain of Grb7 involved in cancer progression. The structure of G7-18NATE bound to the Grb7-SH2 domain previously revealed the close proximity of residues 1 and 8 in the 11-residue cyclic peptide leading to the rational strategy of tethering these residues to constrain the peptide to its bound conformation. While a disulphide tether did not result in a bicyclic peptide with enhanced affinity, the G7-B1 peptide, formed with an O-allylserine-based olefin staple, possessed 2–3 fold increased affinity for the target over G7-18NATE^29^. The current work was thus carried out to determine the structural basis for the improved affinity of the G7-B1 peptide compared to G7-18NATE and to utilize this information for subsequent design of peptides with further improved affinity for the target.

Unexpectedly the crystal structure of the G7-B1 bound to Grb7-SH2 domain revealed that the bicyclic peptide was bound to the Grb7-SH2 domain in an alternative binding conformation to that adopted by G7-18NATE. Rather than just acting as a tether, the staple formed new contacts at the surface of the protein, displacing contacts previously made by residues 9, 10 and 11. G7-B1 residues 2–7 remained in their expected position bound at the pY binding site of the Grb7-SH2 domain, analogous to their mode of binding in G7-18NATE, though with a few extra interactions facilitated by a phosphate ion, present in the crystallisation conditions. Residues 9, 10 and 11 adopted a loop structure away from the protein binding surface. Thus the enhanced binding observed for G7-B1 was due to the alternative binding mode that this peptide could adopt.

While reports of the use of stapled peptides are rapidly accumulating, there are relatively few determined structures of stapled peptides in complex with their binding targets^35^. Thus the structure and role of the staple in the binding interaction remain largely unconfirmed. In most cases where this has been investigated, the staple performs as anticipated - tethering adjacent regions of the peptide without interacting with the target protein. However, there are a few examples in which the staple group does form additional contacts with the target protein hydrophobic surface^41^,^42^. In these cases the staple does not disrupt the binding mode of the peptide, but contributes alongside the peptide to augment its binding. To our knowledge, there are no reported cases where a staple has formed an alternative interaction to the original binding mode of the peptide ligand as seen here.

This observation led to the question of whether the displaced residues F9, P10 and C11 in G7-B1 were still needed for binding to the Grb7-SH2 domain. This was tested with the G7-B1NT peptide in which residues 9–11 were deleted, leaving a monocyclic peptide comprising of residues 1–8 linked by the staple. The G7-B1NT peptide showed a dramatically reduced affinity for the Grb7-SH2 domain compared with G7-B1 demonstrating that, in fact, the additional tether formed by residues 9–11 was important for target binding. This thioether linkage thus potentially constrains the peptide allowing residues 2–8 and the staple to orient in a way that is optimal for the Grb7-SH2 interaction.

This new structural data prompted an investigation of additionally constrained peptides G7-B3 and G7-B4 in which residues F9 and P10 were sequentially deleted. Interesting, G7-B3, in which only F9 was deleted, bound with decreased affinity compared with G7-B1. It is possible that the loss of a β-turn formed by a hydrogen bond between the carbonyl of D6 and the amine of F9 results in the lower affinity measured. In contrast, the G7-B4 peptide, in which both F9 and P10 were deleted, exhibited enhanced binding affinity, potentially reflecting both the improved structural constraint that is imposed on the peptide structure, and the restoration of a β-turn formed by a hydrogen bond to the NH of residue C9.

The G7-B4 peptide thus represents an improved inhibitor of the Grb7-SH2 domain, in terms of both its approximately 2-fold improved affinity over G7-B1 and the removal of unnecessary amino acids. The structure of the G7-B4 peptide was also determined and confirmed that the structural basis for its interaction with the Grb7-SH2 domain was as anticipated. Residues 1–8 and the olefin-based staple adopted the same arrangement as seen for the G7-B1 peptide at the surface of the Grb7-SH2 domain. The thioether linked tether, now consisting only of C9 linked via its sidechain thiol and the N-terminus of residue 1, is a more constrained tether and allows internal hydrogen bond formation between the carbonyl of D6 and the NH of residue 9. The structure showed no new interactions were formed by this shortened linker that could explain the improvement in binding affinity compared to G7-B1. It can be speculated that the improvement is purely though the restraint of the peptide in its bound conformation. The structure of G7-B4NS, which shows reduced binding affinity, was also solved. This confirmed that the binding mode adopted by the peptide is equivalent to that of G7-B4 but that the free O-allylserine sidechains do not form strong interactions with the protein surface. Thus the fully formed staple is established as an important group for the binding of the G7-B4 peptide.

The Grb7-SH2/G7-B complex structures revealed a variety of anions bound at the pY binding site, appearing to augment the binding interaction. In the case of the Grb7-SH2/G7-B1 structure, the anion was phosphate. In the G7-B4/Grb7-SH2 complex it was malonate, and in the G7-B4NS/Grb7-SH2 structure it was phosphate again. In all cases the anions form hydrogen bonds and electrostatic interactions with residues in the BC loop of Grb7, stabilising this loop and thereby facilitating extra interactions between the peptide and protein. This has been observed previously in apo-structures of Grb7-SH2 domain^27^,^31^, but not alongside a G7 peptide bound to the Grb7-SH2 domain. Interestingly, when a peptide is present, the ion does not appear to have any impact on the position of the peptide. In all cases in the current study the peptide backbone at the pTyr binding site is superposable. Together, this reinforces our understanding that the anion can act as a surrogate for the covalently bound phosphate of pY that is the physiological ligand of the SH2 domain. We have recently reported the use of carboxylate-based pY mimetics for enhancing the binding of G7-18NATE to the Grb7-SH2 domain and shown their utility under conditions of physiologically relevant phosphate concentrations^27^. These pY mimetics, used in combination with the G7-B peptides are predicted to enhance the binding affinity even further.

Finally we sought to determine whether the G7-B4 peptide, as one of a new class of Grb7-SH2 ligand, maintains its specificity for Grb7-SH2 over other closely related SH2 domains. This is essential for its use in probing the function of Grb7 in cells or as a potential new targeted therapeutic. We therefore measured the binding affinity of G7-B4 to Grb10- and Grb14-SH2 domains that are the most closely related SH2 domains to Grb7-SH2, and to the Grb2-SH2 domain that shares physiological binding partners with Grb7. G7-B4 shows over 130-fold stronger binding to Grb7-SH2 domain, than these other SH2 domains, demonstrating that the structural improvements made have enhanced the interactions to the Grb7-SH2 domain interaction without enhancing any of the interactions to the other SH2 domains. G7-B4 is thus the highest affinity specific peptide inhibitor of the Grb7-SH2 domain developed to date, with potential as a new lead scaffold for further rational structural improvements to increase the potency of the inhibitor to therapeutic levels.

## Methods

### Preparation of proteins

The pGEX2T plasmids with inserts containing Grb7-SH2 (encoding residues 415–532), Grb2-SH2 (encoding residues 58–160), Grb10-SH2 (encoding residues 471–594) and Grb14-SH2 (encoding residues 426–540) were kindly provided by Dr. Roger Daly. Glutathion-S-Transferase (GST)-Grb2-SH2, GST-Grb7-SH2, Grb-10-SH2 and GST-Grb14-SH2 were all expressed and purified as previously described^26^. Briefly the proteins were overexpressed in *E. coli* strain BL21 (DE3) pLysS. GST fusion protein used in SPR studies were purified by glutathione affinity chromatography, followed by size exclusion chromatography. The free Grb7-SH2 used in crystallography was expressed as a GST-fusion protein and was purified by GST affinity, followed by thrombin cleavage to liberate free Grb7-SH2, and then purified by cation exchange chromatography, and size exclusion chromatography as previously described^43^. The concentrations of GST-Grb2-SH2, GST-Grb7-SH2, SHT-Grb10-SH2, GST-Grb14-SH2 and Grb7-SH2 were determined spectrophotometrically at 280 nm using extinction coefficients of 58 330, 51 340, 49 850, 51 340, and 8480 M^−1^cm ^−1^ respectively^44^.

### Synthesis of peptides

Details for the synthesis of all peptides used in this study are supplied as Supplementary Information. In brief, Bicyclic peptide G7-B1 (cyclo-(CH_2_CO-(XFEGYDNXFPC)-CONH_2_ where X = O-allylserine), was prepared by solid phase synthesis as a peptide amide using Fmoc chemistry on a rink amide resin and ring closed via O-allyl serine residues, cleaved and cyclized by a thioether bond, as previously described^29^.

Monocyclic peptide G7-B1NT (CH_3_-CO-NH_2_-(XFEGYDNX)-CONH_2_ where X = O-allylserine) was prepared by solid phase synthesis as a peptide amide using Fmoc chemistry on rink amide resin. All Fmoc-amino acids were commercially supplied except for Fmoc-O-allylserine that was synthesised in-house using previously established methods^45^. After removing the terminal Fmoc protecting group on the peptide, the resin was treated with acetic anhydride/DIPEA/DMF to afford an acetyl-capped N-terminus. Ring closing metathesis was performed in solution using Horveyda-Grubbs II generation catalyst. The stapled peptide was purified to homogeneity using rpHPLC and its identity confirmed using mass spectrometry (Calculated m/z (C_45_H_57_N_10_O_17_)^−^: 1009.4, Found (C_45_H_57_N_10_O_17_)^−^: 1009.3).

Bicyclic peptide G7-B3 (cyclo-(CH_2_CO-(XFEGYDNXPC)-CONH_2_ where X = O-allylserine) and bicyclic peptide G7-B4 (cyclo-(CH_2_CO-(XFEGYDNXC)-CONH_2_ where X = O-allylserine) cyclized via thioether bond and ring closed via O-allylserines were prepared by solid phase synthesis as peptide amides using Fmoc chemistry on a rink amide resin. After removing the N-terminal Fmoc group the resin was treated with chloroacetic anhydride/DIPEA/DMF to afford a chloroacetyl-capped N-terminus. After peptide cleavage from the resin thioether formation was performed on the crude peptide under basic aqueous conditions then purified using rpHPLC prior to the second cyclisation step. Ring closing metathesis (RCM) was performed in solution, using Hoveyda-Grubbs II generation catalyst. A final purification step using rpHPLC afforded pure peptides as verified using mass spectrometry (G7-B3: Calculated m/z (C_53_H_67_N_12_O_19_S)^−^: 1207.4, Found (C_53_H_67_N_12_O_19_S)^−^: 1207.3; G7-B4: Calculated m/z (C_48_H_60_N_11_O_18_S)^−^: 1110.4 Found (C_48_H_60_N_11_O_18_S)^−^: 1110.3).

Monocyclic peptide G7-B4NS (cyclo-(CH_2_CO-(XFEGYDNXC)-CONH_2_ where X = O-allylserine) cyclized by a thioether bond but without performing ring closing metathesis was synthesized as for G7-B4. A final purification step using rpHPLC afforded pure peptide as verified using mass spectrometry (G7-B4NS: Calculated m/z (C_50_H_64_N_11_O_18_S)^−^: 1138.4; Found (C_50_H_64_N_11_O_18_S)^−^: 1138.1).

Peptide concentrations were determined spectrophotometrically at 280 nm using an extinction coefficient of 1490 M^−1 ^cm^−1^ for all peptides^44^.

### Protein crystallization, X-ray diffraction data collection and structure determination

The crystallization methodology and diffraction data collection for the Grb7-SH2/G7-B1 complex have been reported previously^30^. X-ray diffraction data were collected on the microfocus beamline (MX2) at the Australian Synchrotron using an ADSC Quantum 315r detector and the BLU-ICE software for data acquisition^46^. The diffraction data were indexed and integrated using IMOSFLM^47^ and scaled using AIMLESS^48^ from the CCP4 suite^49^. MOLREP^50^ was used for molecular replacement with one chain of the apo Grb7-SH2 domain (PDB ID: 2QMS) used as the search model. Iterative rounds of structure refinement and model building were carried out using PHENIX^51^ and COOT^52^.

To generate crystals of the Grb7-SH2/ G7-B4 complex, the Grb7-SH2 domain was concentrated to 10.7 mg/mL (784 μM) in 50 mM MES pH 6.6, 100 mM NaCl, and 1 mM DTT and added to lyophilized G7-B4 to achieve a 1:1.5 M protein to peptide ratio. Complex crystals formed over 3 days in a 2 μL hanging drop using 5% (*v/v*) Tacsimate pH 5.0 and 8% (*w/v*) PEG 3350 as the precipitant. Harvestable crystals were cyroprotected with mother liquor supplemented with 20% (*v/v*) glycerol prior to flash-freezing in liquid nitrogen. X-ray diffraction data were collected at the Australian Synchrotron as described above. The collected diffraction images were indexed, integrated and scaled with the software pipeline XIA2^53^,^54^,^55^. MOLREP^50^ from the CCP4 suite was used for molecular replacement with one chain of the apo Grb7-SH2 domain structure (PDB ID: 4WWQ) used as the search model. Model building and refinement were carried out as for the Grb7-SH2/G7-B1 structure. Restraint files for G7-B4 were generated from SMILES strings using phenix.elbow^56^.

The Grb7-SH2/G7-B4NS complex was formed by combining Grb7-SH2 (784 μM) in 50 mM MES pH 6.6, 100 mM NaCl, and 1 mM DTT with lyophilized G7-B4NS to achieve a 1:1.5 M ratio of protein to peptide. Crystals of the complex formed over 3 days in a 0.2 μL sitting drop using 0.2 M sodium fluoride and 20% (*w/v*) PEG 3350 as the precipitant. X-ray diffraction data were collected on the MX2 beamline at the Australian Synchrotron as per the previous structures. The diffraction images were indexed and integrated using IMOSFLM^47^ and scaled using AIMLESS^48^ from the CCP4 suite^49^. Molecular replaement, model building and refinement were carried out as for the Grb7-SH2/G7-B1 structure.

MOLPROBITY^57^ was used to assess the quality of the final model and figure were generated using PyMOL. A summary of the crystallographic information, including data collection, processing and refinement statistics, is provided in Table 1.

### Surface Plasmon Resonance

Surface Plasmon resonance experiments were performed using a BIAcore T100. Polyclonal rabbit anti-GST antibody (Abcam, Cambridge, UK) was immobilized on the active and reference cell of a BIAcore CM5 series S sensor chip (GE Life Science) using an amine coupling kit (GE Life Science). Immobilisation levels for the anti-GST antibody were 1 × 10^5^ RU. GST-Grb2-SH2, GST-Grb7-SH2, Grb-10-SH2 and GST-Grb14-SH2 were immobilized on the active cells while recombinant GST was immobilized on the control cells by injecting each protein at a concentration of 0.7 μM as previously described^33^. Immobilisation levels for GST-Grb2-SH2, GST-Grb7-SH2, Grb-10-SH2 and GST-Grb14-SH2 were all 2 × 10^3^ RU while GST immobilisation levels were 1 × 10^3^ RU. Triplicate samples of G7-B3, G7-B4, G7-B4NS and G7-B1NT in Running Buffer (50 mM sodium phosphate, 150 mM NaCl, 1 mM DTT, pH 7.4) at concentrations of 0–100 μM were injected for 60 sec at a flow rate of 30 μL/min, with a dissociation time of 3 min. Sensorgrams from triplicate runs were superposable. The experimental temperature was 25 ^o^C. Data was analyzed using Scrubber2 (BiaLogic Software, Campbell, ACT, Australia) and SigmaPlot (Systat Software Inc). Reported standard deviations for *K*_D_ values were based on errors associated with concentration determination.

## Additional Information

**Accession numbers**: Atomic coordinates and structure factors have been deposited in the RCSB PDB under the accession numbers PDB: 5EEQ, 5EEL and 5D0J.

**How to cite this article**: Gunzburg, M. J. *et al.* Unexpected involvement of staple leads to redesign of selective bicyclic peptide inhibitor of Grb7. *Sci. Rep.* **6**, 27060; doi: 10.1038/srep27060 (2016).

## Supplementary Material

Supplementary Information

## Figures and Tables

**Table 1:** Summary of crystallographic information.

Data collection	Grb7-SH2/G7-B1	Grb7-SH2/G7-B4	Grb7-SH2/G7-B4NS
Wavelength	0.954	0.954	0.954
Space group	*P* 2_1_	*P* 4_1_2_1_2	*P* 2_1_2_1_2_1_
Unit cell dimensions			
*a, b, c* (Å)	37.08, 63.81, 52.04	95.23, 95.23, 241.57	33.98, 94.04, 131.49
α, β, γ (°)	90, 92.5, 90	90, 90, 90	90, 90, 90
Resolution (Å)	40.31-1.6 (1.63-1.6)	46.72-2.47 (2.56-2.47)	38.24-2.6 (2.693-2.6)
†R_merge_ (%)	4.2 (55.7)	11.12 (67.35)	3.85 (15.81)
I/σI	13.3 (1.8)	16.84 (3.84)	10.98 (4.08)
Unique reflections measured	31980 (1577)	40842 (3996)	13546 (1332)
Completeness (%)	99.80 (100.00)	99.97 (99.73)	99.19 (99.85)
Multiplicity	3.5 (3.5)	12.9 (12.5)	2.0 (2.0)
Refinement			
R_work_ (%)	17.34 (28.63)	18.37 (24.07)	18.69 (26.60)
R_free_ (%)	19.61 (30.31)	23.12 (30.15)	24.57 (33.50)
No. of atoms			
Macromolecules	1822	5364	3220
Ligands	38	140	10
Solvent	170	75	11
Mean B-factors (Å^2^)			
Macromolecules	35.00	46.60	41.30
Ligands	41.60	45.90	45.60
Solvent	44.60	39.40	36.30
RMSDs			
Bond lengths (Å)	0.005	0.007	0.007
Bond angles (°)	0.93	1.04	1.09
Ramachandran plot (%)			
Favoured regions	100	99	99
Allowed regions		1	1

^†^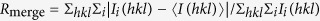. where *I*_*i*_(*hkl*) is the *i*th intensity measurement of reflection 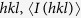 its average. Values given in parantheses, are for the high resolution shell.

**Table 2:** Binding parameters for peptides binding Grb SH2 domains.

Peptide	SH2 protein	*K*_*D*_^a^ (μM)	*B*_ma_^b^ (RU)	Theoretical *B*_max_
G7-B1	Grb7	1.50 ± 0.01	23.78 ± 0.03	38
G7-B1NT	Grb7	ND^c^	ND^c^	68
G7-B3	Grb7	4.90 ± 0.03	53.47 ± 0.02	82
G7-B4	Grb7	0.83 ± 0.006	40.78 ± 0.03	59
G7-B4NS	Grb7	4.93 ± 0.03	34.78 ± 0.01	55
G7-B4	Grb2	ND^c^	ND^c^	54
G7-B4	Grb10	ND^c^	ND^c^	61
G7-B4	Grb14	ND^c^	ND^c^	45

^a^*K*_D_ (equilibrium dissociation constant) was derived from fits to a single-site saturation model. Errors are standard deviations based on errors arising from concentration determination.

^b^B_max_ (maximum binding signal) was derived from fits to single-site saturation model. Errors are standard errors arising from fits.

^c ^Not determined, as the affinity was too weak to reliably fit the data.
