# Supplementary material for: Unexpected involvement of staple leads to redesign of selective bicyclic peptide inhibitor of Grb7
Source: Sci Rep. 2016 Jun 3;6:27060. doi: 10.1038/srep27060 (PMC4891710; doi:10.1038/srep27060)
Supplement: Supplementary Information [file srep27060-s1.pdf]

Supplementary Experimental Information

I. Synthesis of monocyclic peptide G7-B1NT

G7-B1NT **(CH_3-CONH-(XFEGYDNX)-$CONH_2$ where X=*O*-allyl serine),** ring closed via *O*-allyl-serines, was synthesised on a 0.25 mmol scale using standard Fmoc chemistry on a Rink amide resin (~0.7 mmol/g loading). The Rink amide resin (350 mg, 0.25 mmol) was washed with DMF (2 x 4 mL). The Fmoc protecting group on the resin was removed with 2 washes of 20 % piperidine, with 0.1 M HOBt, in DMF (4 mL) for 15 min each. The resin was thoroughly washed with DMF (3 x 4 mL) and then soaked in Fmoc-protected amino acid (3.1 eq. to resin loading), dissolved in DMF (4 mL) along with HBTU (3 eq. to resin loading), HOBt (3 eq. to resin loading) and DIPEA (4.5 eq. to resin loading), for 45 min. The amino acid coupling cycle was then repeated. The resin was washed with DMF (3 x 5 mL), and the Kaiser test was performed on a few beads of the resin to confirm complete coupling. The terminal Fmoc protecting group on the amino acid was removed with 2 washes of 20 % piperidine, with 0.1 M HOBt, in DMF (4 mL) for 15 min each. The peptide elongation cycle was then repeated until the peptide sequence was complete. After removing the terminal Fmoc protecting group on the peptide, the resin was treated twice with 10 % v/v acetic anhydride and 1 % v/v DIPEA in DMF (4 mL), for 20 min each, to afford an acetyl-capped N-terminus. The resin was subsequently washed with DMF (3 x 4 mL), CH_2Cl_2 (2 x 4 mL), Et_2O (3 x 4 mL), and air dried for 20 min. Cleavage was performed by treating the resin with a cleavage solution (10 mL) comprising of 250 µL of distilled water (2.5 % v/v), 250 µL of triisopropylsilane (2.5 % v/v), 50 µL of ethanedithiol (0.5 % v/v) in TFA, for 2.5 h. TFA was then evaporated under a stream of N_2 and the peptide was precipitated by addition of Et_2O (50 mL). The precipitate was filtered and redissolved in 50% aqueous CH_3CN for lyophilisation.

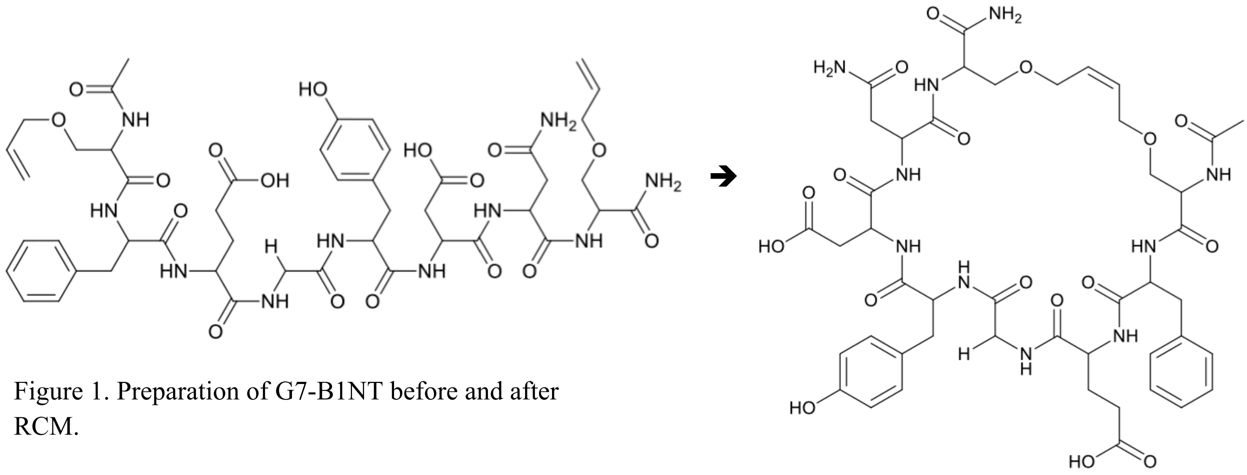

Figure 1. Preparation of G7-B1NT before and after RCM.

Ring closing metathesis (RCM) of the acetyl capped peptide (30 mg, 28.9 µmol) was performed in solution, using 2,2,2,-trifluoroethanol and CH_2Cl_2 as the solvent in 4:1 ratio (3 mL), at approximately 10 mM concentration, with Hoveyda-Grubbs II generation as the catalyst (35 mol %, 6.3 mg), under microwave irradiation, for 3 h at 65 ºC. Solvent was then evaporated under reduced pressure and the crude peptide was purified using a Hewlett-Packard 1200 series HPLC system (Agilent Technologies, CA), by injecting the sample onto a reverse-phase preparative (C18, 300 Å, 5 µm, 10 mm x 250 mm) column, eluted over a 50 min gradient from 0 to 60 % solvent B, (solvent A: 0.1 % TFA/H_2O; solvent B: 0.1 % TFA/CH_3CN) with a flow rate of 6 mL/min. The fractions were collected and then analysed with liquid chromatography and mass spectrometry. The pure fractions were lyophilised to afford the cyclic peptide.

Purified peptide was analysed using a Hewlett-Packard 1100 series HPLC system (Agilent Technologies, CA). The samples were injected onto a reverse-phase Vydac™ analytical (C18, 300 Å, 5 µm, 4.6 mm x 150 mm) column and eluted over a 45 min gradient from 0 to 50 % solvent B, (solvent A: 0.1 % TFA/H_2O; solvent B: 0.1 % TFA/CH_3CN) with a flow rate of 1 mL/min. The analytical trace confirmed that the peptide was purified to > 95% homogeneity. The mass spectrum was acquired using an Agilent 1100 MSD SL ion trap mass spectrometer confirming its identity.) (Calculated m/z $(C_{45}H_{57}N_{10}O_{17})^-$:1009.4, Found $(C_{45}H_{57}N_{10}O_{17})^-$: 1009.3

II. Synthesis of bicyclic peptides G7-B3 and G7-B4

Bicyclic peptide G7-B3 (cyclo-(CH_2CONH-(XFEGYDNXPC)-$CONH_2$ where X=*O*-allyl-serine) and bicyclic peptide G7-B4 (cyclo-(CH_2CONH-(XFEGYDNXC)-$CONH_2$ where X=*O*-allyl-serine) cyclized by head-to-tail thioether bond and ring closed via *O*-allyl-serines were each synthesised on a 0.25 mmol scale using standard Fmoc chemistry on a Rink amide resin (0.7 mmol/g loading). The Rink amide resin (350 mg, 0.25 mmol) was washed with DMF (2 x 4 mL). The Fmoc protecting group on the resin was removed with 2 washes of 20 % piperidine, with 0.1 M HOBt, in DMF (4 mL) for 15 min each. The resin was thoroughly washed with DMF (3 x 4 mL) and then soaked in Fmoc-protected amino acid (3.1 eq. to resin loading), dissolved in DMF (4 mL) along with HBTU (3 eq. to resin loading), HOBt (3 eq. to resin loading) and DIPEA (4.5 eq. to resin loading), for 45 min. The amino acid coupling cycle was then repeated. The resin was washed with DMF (3 x 4 mL), and the Kaiser test was performed on a few beads of the resin to confirm complete coupling. The terminal Fmoc protecting group on the amino acid was removed with 2 washes of 20 % piperidine, with 0.1 M HOBt, in DMF (4 mL) for 15 min each. The peptide elongation cycle was then repeated until the peptide sequence was complete. After removing the terminal Fmoc protecting group on the peptide, the resin was treated with chloroacetic anhydride (171 mg, 1 mmol) and DIPEA (100 μL) in DMF (2 mL) for 30 min to afford a chloroacetyl-capped N-terminus. The resin was subsequently washed with DMF (3 x 4 mL), CH_2Cl_2 (2 x 4 mL), Et_2O (2 x 4 mL), and air dried for 20 min. Cleavage was performed on 0.3 mmol of the resin, by treating the resin with a cleavage solution (10 mL) comprising of 250 μL of distilled water (2.5 % v/v), 250 μL of triisopropylsilane (2.5 % v/v), 50 μL of ethanedithiol (0.5 % v/v) in TFA, for 2.5 h. TFA was then evaporated under a stream of N_2 and the peptide was precipitated by addition of Et_2O (50 mL). The precipitate was filtered and redissolved in 50% aqueous CH_3CN for lyophilisation.

After lyophilisation, thioether formation was effected by dissolving the peptide (~2 mg/mL concentration) in 50 mM NH_4HCO_3 solution (made up in 50% aqueous CH_3CN), for 1.5 h at room temperature. The success of cyclisation was confirmed using an Agilent 1100 MSD SL ion trap mass spectrometer. The cyclised peptide was then lyophilised and purified using a Hewlett-Packard 1200 series HPLC system (Agilent Technologies, CA), by injecting the sample onto a reverse-phase preparative (C18, 300 Å, 5 μm, 10 mm x 250 mm) column, eluted over a 50 min gradient from 0 to 60 % solvent B, (solvent A: 0.1 % TFA/H_2O; solvent B: 0.1 % TFA/CH_3CN) with a flow rate of 6 mL/min. The fractions were collected and then analysed with liquid chromatography and mass spectrometry. The pure fractions were lyophilised to afford the cyclised peptide.

Ring closing metathesis (RCM) of the cyclised peptide (34 mg, 27.5 μmol for G7-B3 and 30 mg, 26.3 μmol for G7-B4) was performed in solution, using 2,2,2,-trifluoroethanol and CH_2Cl_2 as the solvent in 4:1 ratio (3 mL), at approximately 10 mM concentration, with Hoveyda-Grubbs II generation as the catalyst (35 mol %, under microwave irradiation, for 3 h at 65 ºC. Solvent was then evaporated under reduced pressure and the crude peptide was purified using a Hewlett-Packard 1200 series HPLC system (Agilent Technologies, CA), by injecting the sample onto a reverse-phase preparative (C18, 300 Å, 5 μm, 10 mm x 250 mm) column, eluted over a 50 min gradient from 0 to 60 % solvent B, (solvent A: 0.1 % TFA/H_2O; solvent B: 0.1 % TFA/CH_3CN) with a flow rate of 6 mL/min. The fractions were collected and then analysed with liquid chromatography and mass spectrometry. The pure fractions were lyophilised to afford the bicyclic peptide.

Purified peptide was analysed using a Hewlett-Packard 1100 series HPLC system (Agilent Technologies, CA). The samples were injected onto a reverse-phase Vydac™ analytical (C18, 300 Å, 5 μm, 4.6 mm x 150 mm) column and eluted over a 45 min gradient from 0 to 50 % solvent B, (solvent A: 0.1 % TFA/H_2O; solvent B: 0.1 % TFA/ CH_3CN) with a flow rate of 1 mL/min. The analytical traces confirmed that the peptides were purified to > 95% homogeneity. The mass spectrum was acquired using an Agilent 1100 MSD SL ion trap mass spectrometer confirming the identity of the peptides. (G7-B3:Calculated $(C_{53}H_{67}N_{12}O_{19}S)^-$: 1207.4, Found $(C_{53}H_{67}N_{12}O_{19}S)^-$: 1207.3; G7-B4: Calculated $(C_{48}H_{60}N_{11}O_{18}S)^-$: 1110.4 Found $(C_{48}H_{60}N_{11}O_{18}S)^-$: 1110.3

III. Synthesis of monocyclic peptides G7-B4NS

Monocyclic peptide G7-B4NS (cyclo-(CH_2CONH-(XFEGYDNXC)-$CONH_2$ where X=*O*-allyl serine) cyclized by head-to-tail thioether bond but without cyclizing the *O*-allyl-serine tethers was synthesized as per G7-B4, but without the RCM step. Analytical rpHPLC traces confirmed that the peptide was purified to > 95% homogeneity. The mass spectrum was acquired using an Agilent 1100 MSD SL ion trap mass spectrometer confirming the identity of the peptides. Calculated $(C_{50}H_{64}N_{11}O_{18}S)^-$: 1138.4; Found $(C_{50}H_{64}N_{11}O_{18}S)^-$: 1138.1

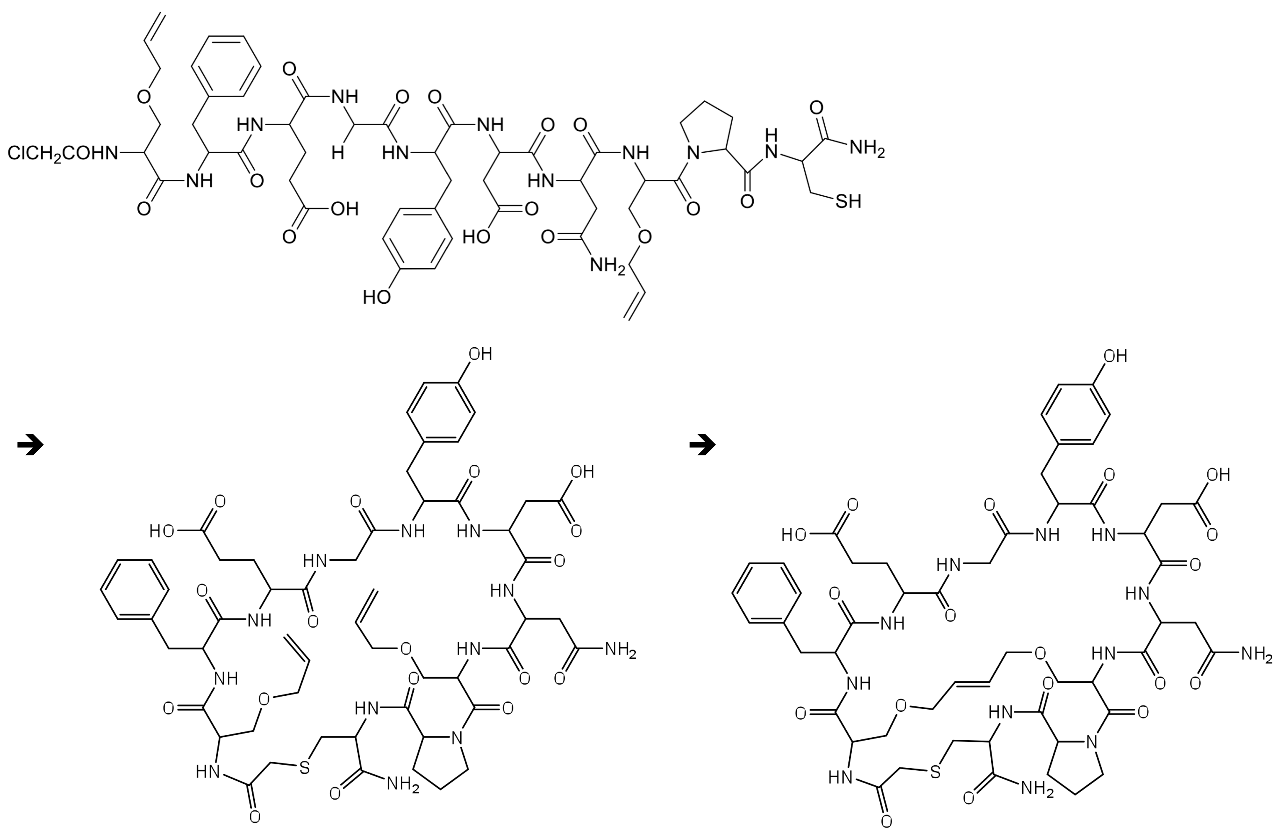

Figure 2. Preparation of G7-B3 showing thioether formation and ring closure.

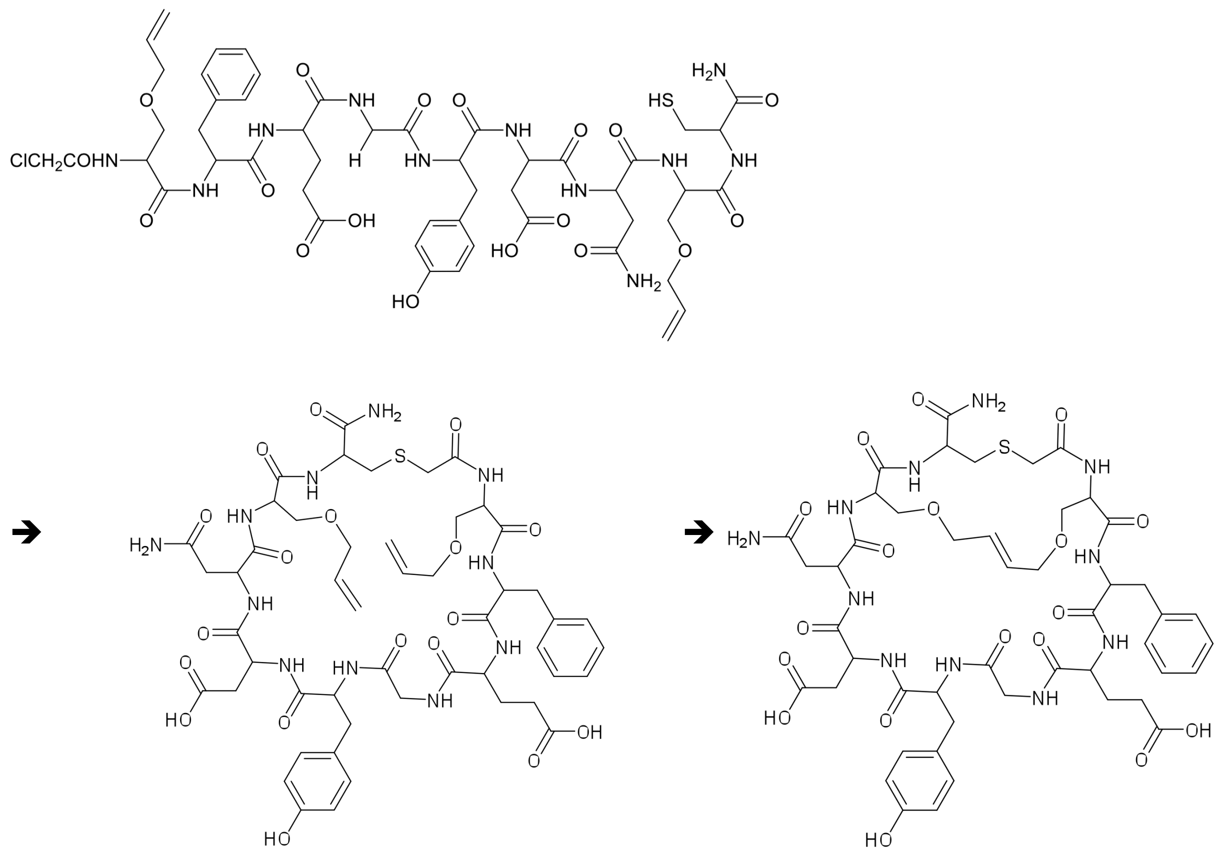

Figure 3. Preparation of G7-B4 showing thioether formation and ring closure.
